# Anesthetic Management of a Pediatric Patient With CTNNB1-Related Neurodevelopmental Disorder: A Case Report

**DOI:** 10.7759/cureus.112723

**Published:** 2026-07-15

**Authors:** Ana Rita Rocha, Conceição Cardante Santos, Petra Alves, Cristina Peixoto Gomes

**Affiliations:** 1 Anesthesiology and Perioperative Medicine, Unidade Local de Saúde de Trás-os-Montes e Alto Douro, Vila Real, PRT; 2 Anesthesiology and Perioperative Medicine, Unidade Local de Saúde de Braga, Braga, PRT

**Keywords:** ambulatory surgery, ctnnb1-related neurodevelopmental disorder, nedsdv, pediatric anesthesia, rare genetic disorder

## Abstract

CTNNB1-related neurodevelopmental disorder with spastic diplegia and visual defect (NEDSDV) is a rare genetic condition characterized by developmental delay, axial hypotonia, peripheral spasticity, and ophthalmologic abnormalities. Due to its phenotypic overlap with bilateral spastic cerebral palsy, NEDSDV may be underdiagnosed, and its anesthetic implications remain undocumented. We report the perioperative management of a two-year-old female with NEDSDV undergoing elective adenoidectomy and myringotomy in a hospital-based ambulatory surgical center. Anesthetic care was individualized, with emphasis on airway planning, cautious titration of anesthetic agents, and multimodal opioid-sparing analgesia. The perioperative course was uneventful, and the patient was safely discharged on the same day. Following a comprehensive literature review, no prior anesthetic reports were identified in patients with NEDSDV. To our knowledge, this case represents one of the first published anesthetic descriptions in a pediatric patient with NEDSDV and highlights key considerations for safe and effective anesthesia in this emerging clinical population.

## Introduction

Neurodevelopmental disorder with spastic diplegia and visual defect (NEDSDV) is a rare autosomal dominant condition caused by pathogenic variants in the CTNNB1 gene, which encodes β-catenin, a critical regulator of synaptic stability and neuronal signaling. The disease mechanism is thought to be haploinsufficiency of β-catenin, disrupting canonical Wnt signaling and impairing synaptic plasticity and neuronal connectivity [1]. Since its first description in 2012, NEDSDV has been increasingly recognized as a differential diagnosis for bilateral spastic cerebral palsy, particularly the traditional phenotype of spastic diplegia, with an estimated prevalence of 2.6-3.2 per 100,000 live births [2].

The phenotype ranges from mild to profound global developmental delay and cognitive impairment. Common features include craniofacial dysmorphisms (long and/or flat philtrum, thin upper lip, broad nasal tip, and small alae nasi), microcephaly, neurological manifestations (truncal hypotonia, peripheral spasticity, and dystonia), and ophthalmologic abnormalities (exudative vitreoretinopathy, strabismus, and refractive errors). Musculoskeletal anomalies, congenital heart defects, and feeding difficulties may also be present [3-4].

These features present specific anesthetic challenges in pediatric patients, including difficult airway management and potentially altered responses to anesthetic agents secondary to neurological dysfunction. Despite advances in the genetic and phenotypic characterization of NEDSDV, there is a notable lack of literature addressing anesthetic considerations for these patients. Anesthetic principles established for other neurodevelopmental disorders, such as cerebral palsy, offer the closest available clinical context, and this report extends such principles to a previously uncharacterized condition [5].

NEDSDV is being increasingly diagnosed, is multisystemic in its presentation, and remains anesthetically uncharacterized. This report details the perioperative anesthetic management of a pediatric patient with NEDSDV, offering one of the first clinical frameworks to guide perioperative decision-making in this rare condition.

## Case presentation

A two-year-old female with genetically confirmed NEDSDV was scheduled for elective adenoidectomy and bilateral myringotomy in an ambulatory surgery setting. The patient exhibited severe psychomotor delay, axial hypotonia with peripheral hypertonia, dystonia, and strabismus. An echocardiogram performed at two months of age revealed a patent foramen ovale, with all other findings within normal limits for her age. Physical status was classified as American Society of Anesthesiologists (ASA) III. Her regular medication regimen included levodopa, melatonin, and vitamin D and was maintained. A previous inhalational anesthesia with sevoflurane for ocular botulinum toxin injection was documented without complications.

Preoperative evaluation revealed microcephaly, mild retrognathia, high-arched palate, and relative macroglossia. Cardiopulmonary auscultation was normal. Anthropometric measurements revealed a weight of 10.5 kg and a height of 82 cm. Laboratory evaluation, including complete blood count, serum electrolytes, coagulation studies, and renal function tests, revealed mild hyperkalemia (5.3 mmol/L), with all other parameters within normal limits (Table 1). After a comprehensive discussion of anesthetic risks with the family, informed consent was obtained for general anesthesia.

**Table 1:** Preoperative laboratory assessment. Note: Reference ranges are from the laboratories of the author's institution.

Variable		Value	References ranges
Basic metabolic panel	Chloride (mmol/L)	107	98-107
	Creatinine (mg/dL)	0.3	0.24-0.41
	Potassium (mmol/L)	5.3	3.5-5.1
	Sodium (mmol/L)	138	136-145
	Urea (mg/dL)	32	19-49
Coagulation profile	Activated partial thromboplastin time (sec)	32.2	25.0-37.0
	Prothrombin time (sec)	12.9	8.0-14.0
Complete blood count	White blood cells (10^3^/uL)	8.7	5.0-14.5
	Haemoglobin (g/dL)	12.8	11.5-13.5
	Platelets (10^3^/uL)	269	200-450

On the day of surgery, the anesthesia workstation was accordingly prepared, and a pediatric difficult airway cart was immediately available in the operating room. Monitoring included ASA standards I and II with temperature assessment and cerebral oxygenation monitoring using near-infrared spectroscopy (INVOS™, Medtronic, Minneapolis, Minnesota).

Anesthesia was induced using inhalational sevoflurane (5% in 100% oxygen) to facilitate intravenous cannulation. During induction, the patient developed transient apnea at end-tidal sevoflurane concentration of 1.8%. Adequate oxygenation was maintained with mask ventilation without airway adjuncts. After establishing a 22-gauge peripheral venous access, anesthesia was deepened with intravenous fentanyl (2 mcg/kg) and propofol (2 mg/kg). A size 2 armored laryngeal mask airway (LMA) was successfully placed on the first attempt. Maintenance with sevoflurane was titrated according to age-adjusted MAC and standard clinical parameters, with end-tidal concentrations maintained between 1.5% and 2.0%.

Intraoperative analgesia included paracetamol (15 mg/kg) and tramadol (2 mg/kg). Dexamethasone (0.15 mg/kg) and ondansetron (0.1 mg/kg) were administered for antiemetic prophylaxis. Surgery lasted 40 minutes without anesthetic or surgical complications.

The LMA was removed under deep anesthesia while the patient maintained spontaneous ventilation with adequate oxygenation and normocapnia. Emergence was uneventful, with no airway complications or signs of respiratory distress.

The patient completed both Phases I and II recovery without complications. After meeting discharge criteria and comprehensive family education, the patient was discharged home on the same day. Instructions included information on warning signs and direct contact details for the Ambulatory Unit. Postoperative analgesia with oral paracetamol was prescribed to ensure adequate pain control at home.

## Discussion

This case highlights the perioperative management of a pediatric patient with NEDSDV undergoing ambulatory otolaryngologic surgery. Due to the rarity of this condition and absence of anesthetic-specific literature, our perioperative strategy was guided by established approaches to patients with complex neurodevelopmental profiles and by careful consideration of the patient’s individual phenotype.

CTNNB1-related disorders present with central hypotonia, peripheral spasticity, dystonia, and ophthalmologic abnormalities, often accompanied by cognitive and communication impairments [3]. Efforts were made to establish a therapeutic connection with the child by using communication strategies adapted to her cognitive abilities. Parental involvement supported cooperation and helped mitigate preoperative anxiety. These measures improved compliance with monitoring and reduced the risk of distress during induction or emergence.

Recent studies suggest that the CTNNB1 gene, via Wnt/β-catenin signaling, contributes not only to central nervous system development but also to cardiac morphogenesis. Sinibaldi et al. (2023) reported congenital heart defects in approximately 26% of affected patients, including structural anomalies such as tetralogy of Fallot, mitral valve prolapse, and atrioventricular septal defects [4]. Although our patient exhibited no cardiac symptoms, the multisystemic nature of the disorder warranted a comprehensive preoperative cardiac assessment, which excluded clinically relevant cardiac abnormalities. This emphasizes the need for systematic cardiovascular screening in these patients, even when asymptomatic.

In our patient, the presence of craniofacial abnormalities raised concerns about potential risk for airway difficulties. Although airway management was successfully achieved with an LMA, a difficult airway cart was readily available, and both induction and emergence from anesthesia were conducted with a structured difficult airway management plan in place.

Although no data exist on anesthetic sensitivity in NEDSDV, the transient apnea observed during low-dose sevoflurane induction in this case highlights the need for cautious titration. The etiology of this event remains unclear and may reflect an individual sensitivity rather than a syndrome-specific trait. However, given the potential variability in anesthetic responses among patients with other neurodevelopmental conditions, careful monitoring and tailored anesthetic dosing remain essential to ensure safety [5].

Processed EEG-based depth monitoring was not employed given its documented limited reliability in children under two years of age [6]. Regional cerebral oxygen saturation (rSO₂) was additionally assessed using near-infrared spectroscopy (INVOS™, Medtronic, Minneapolis, Minnesota). In the context of NEDSDV, where the complexity of the underlying neurological phenotype may be associated with atypical physiological responses during anesthesia, continuous rSO₂ monitoring was employed as an adjunctive safety measure to complement standard hemodynamic parameters [7].

Neuromuscular blockers were not administered, as the nature of the procedure did not require complete neuromuscular relaxation. Although the neuromuscular junction is not known to be directly affected in NEDSDV, patients with other neurodevelopmental disorders, such as cerebral palsy, have shown variable responses to neuromuscular blocking agents, likely due to altered physiology. [8] In this case, avoidance of muscle relaxants may have contributed to an enhanced postoperative recovery by minimizing the risk of residual neuromuscular blockade and respiratory complications.

Analgesic management followed a multimodal, opioid-sparing strategy. The combination of paracetamol, tramadol, and dexamethasone provided effective early postoperative analgesia, reducing the need for stronger opioid agents. This approach is particularly beneficial in children with neurologic impairment, who are more vulnerable to opioid-induced respiratory depression and excessive sedation due to underlying central nervous system dysfunction [9].

Despite an ASA III classification and complex phenotype, the perioperative course was uneventful, and same-day discharge was achieved in accordance with standard pediatric ambulatory criteria. Importantly, the procedure was performed in a hospital-based ambulatory surgical center, ensuring immediate access to advanced pediatric and anesthetic care in the event of intraoperative or postoperative complications. This setting provided an additional layer of safety and was a decisive factor in proceeding with ambulatory management for a high-risk patient.

A comprehensive literature search was conducted in June 2026 across PubMed, MEDLINE, EMBASE, and Google Scholar, combining the search terms "CTNNB1," "NEDSDV," "anesthesia," "anaesthesia," and "perioperative management," with no date or language restrictions. No published reports addressing anesthetic management in patients with NEDSDV were identified. To our knowledge, this is one of the first reports describing anesthetic management in a patient with NEDSDV. As genetic testing becomes more widely available and syndromic diagnoses such as CTNNB1-related disorders are increasingly recognized, anesthesiologists are likely to encounter similar cases in routine practice. This report emphasizes the value of individualized care based on phenotypic expression rather than syndrome name alone and illustrates how standard pediatric anesthetic practices can be adapted safely and effectively.

As a single-case experience, our findings cannot be generalized to the broader NEDSDV population and did not include a standardized functional assessment or extended follow-up. Further case reports with longer follow-up are needed to confirm these observations and inform future perioperative recommendations.

## Conclusions

CTNNB1-related neurodevelopmental disorder is a rare condition with no previously published anesthetic case reports. Early genetic diagnosis enabled tailored perioperative planning and risk mitigation, including airway planning and cautious anesthetic titration. This case illustrates that with individualized management and appropriate resources, general anesthesia can be safely delivered to pediatric patients with NEDSDV in an ambulatory setting.
